# Bortezomib suppresses self‐renewal and leukemogenesis of leukemia stem cell by NF‐ĸB‐dependent inhibition of CDK6 in MLL‐rearranged myeloid leukemia

**DOI:** 10.1111/jcmm.16377

**Published:** 2021-02-17

**Authors:** Bin Zhou, Yaqian Qin, Jingying Zhou, Jichen Ruan, Fang Xiong, Jinglai Dong, Xingzhou Huang, Zhijie Yu, Shenmeng Gao

**Affiliations:** ^1^ Laboratory of Internal Medicine The First Affiliated Hospital of Wenzhou Medical University Wenzhou China; ^2^ Department of Hematology The Second Affiliated Hospital & Yuying Children's Hospital of Wenzhou Medical University Wenzhou China; ^3^ Department of Hematology The First Affiliated Hospital of Wenzhou Medical University Wenzhou China

**Keywords:** Bortezomib, cyclin dependent kinase 6, leukaemia stem cell, MLL rearrangements

## Abstract

Acute myeloid leukaemia (AML) with chromosomal rearrangements involving the H3K4 methyltransferase mixed‐lineage leukaemia (MLL) is an aggressive subtype with low overall survival. Bortezomib (Bort) is first applied in multiple myeloma. However, whether bort possesses anti‐self‐renewal and leukemogenesis of leukaemia stem cell (LSC) in AML with MLL rearrangements is still unclear. Here, we found that bort suppressed cell proliferation and decreased colony formation in human and murine leukaemic blasts. Besides, bort reduced the frequency and function of LSC, inhibited the progression, and extended the overall survival in MLL‐AF9 (MF9) ‐transformed leukaemic mice. Furthermore, bort decreased the percentage of human LSC (CD34^+^CD38^‐^) cells and extended the overall survival in AML blasts‐xenografted NOD/SCID‐IL2Rγ (NSG) mice. Mechanistically, cyclin dependent kinase 6 (*CDK6*) was identified as a bort target by RNA sequencing. Bort reduced the expressions of CDK6 by inhibiting NF ĸB recruitment to the promoter of *CDK6*, leading to the abolishment of NF ĸB DNA‐binding activity for *CDK6* promoter. Overexpression of CDK6 partially rescued bort‐induced anti‐leukemogenesis. Most importantly, bort had little side‐effect against the normal haematological stem and progenitor cell (HSPC) and did not affect CDK6 expression in normal HSPC. In conclusion, our results suggest that bort selectively targets LSC in MLL rearrangements. Bort might be a prospective drug for AML patients bearing MLL rearrangements.

## 1 INTRODUCTION

Acute myeloid leukaemia (AML) is a lethal haematological malignancy with an increasing incidence rate. AML is characterized by stem cell‐like self‐renewal capacity and block of differentiation.^1^ AML blasts have distinct genetic and molecular abnormalities with different overall survival. The mixed‐lineage leukaemia gene (*MLL1* or *KMT2A*) encodes a histone methyltransferase and is essential for maintaining haematopoiesis. About 10% of adult leukaemia and over 70% of infant leukaemia patients carry the translocations of MLL.^2^ The N‐terminal of MLL is fused to over 50 fusion partners, including MLLT3 (also called AF9) and MLLT4 (AF6). The most common translocation is the t(9; 11) (p22; q23) reciprocal translocation, which finally produces the *MLL‐AF9* (*MF9*) fusion gene. Transformation of the murine haematological stem and progenitor cell (HSPC) by *MF9* rapidly induces transplantable leukaemia, which expresses the marker of myeloid lineage.^3^ Additionally, MF9‐transformed leukaemia blasts aberrantly express self‐renewal–associated genes, such as homeobox a9 (*hoxa9*)^4^ and meis homeobox 1 (*meis1*).^5^ Therefore, the critical characteristics of MLL rearrangements are conferring leukaemia‐initiating capability to normal progenitor cells. AML patients with MLL rearrangements are often associated with frequent relapse and poor long‐term survival.^6^ Therefore, new molecular mechanism‐based therapeutic strategies for MLL rearrangements are urgently needed.

Bortezomib (Bort) has been approved as a first‐line drug for multiple myeloma and mantle cell lymphoma by selectively and reversibly inhibiting 26S proteasome.^7^ Treatment of bort accumulates proteins of lysine‐48 ubiquitin in 26S proteasome, resulting in the cytotoxic effects in malignant cells. However, bort exerts comprehensive effects on cancer cells through more complex mechanisms independent of proteasome inhibition. For example, bort induces proteasome‐independent but autophagy‐mediated lysosomal degradation of tumour necrosis factor receptor‐associated factor 6.^8^ The NF ĸB family is comprised of five subunits, including p65, Rel B, c‐Rel, p52, and p50. Constitutive activated NF ĸB signalling is implicated in various types of cancer, angiogenesis, and chronic inflammation.^9^ Bort has been widely used for the therapy of haematological malignancies through suppressing NF ĸB‐dependent transcription.^10^ Therefore, understanding the complicated molecular mechanism by which bort degrades proteins in proteasome‐dependent and ‐independent manner might facilitate the clinical usage of proteasome inhibitors.

Cyclin dependent kinase 6 (CDK6) and its homolog CDK4 are the core components of cell cycle machinery. As a serine/threonine kinase, CDK6 is indispensable for the passage of G1 to S phase by phosphorylating retinoblastoma protein.^11^ Recently, several studies have revealed that in addition to regulating the cell cycle, CDK6 plays essential roles in apoptosis,^12^ reprogramming of cancer cell metabolism,^13^ and self‐renewal ability of leukaemia stem cell (LSC).^14^ For example, CDK6 is an indispensable downstream effector in MLL‐rearranged AML, and knockdown of CDK6 reduces the self‐renewal LSC in MLL arrangements‐transformed mouse leukaemia.^14^ Therefore, CDK6 is a potential and promising target for haematological malignancies. However, whether and how CDK6 is required for bort‐induced anti‐leukemogenesis effect in MLL‐rearranged leukaemia remains to be determined.

In our study, we investigated the anti‐leukemogenesis of bort and found that bort decreased colony number, inhibited the frequencies of LSC, and extended survival time in MF9‐transformed leukaemic mice. Bort decreased the transcript and protein expressions of CDK6 by inhibiting NF‐ĸB recruitment to *CDK6* promoter. Overexpression of CDK6 partially rescued bort‐induced anti‐leukemogenesis ability. Our results describe a new mechanism by which bort suppresses self‐renewal of LSC by NF‐ĸB‐dependent inhibition of CDK6 in MLL‐arranged leukaemia, indicating that bort might be a potential drug for AML patients with MLL rearrangements.

## 2 MATERIAL AND METHODS

### 2.1 Leukaemic cell lines, primary AML blasts, and umbilical cord blood (UCB)

Human leukaemic cell lines THP1 and MV4‐11 (ATCC, Manassas, VA, USA) were used for the present study. All leukaemic cell lines were cultured in a humidified 37°C incubator with 5% CO_2_ in RPMI 1640 (Invitrogen, Carlsbad, CA, USA) with 10% fetal bovine serum (Sigma‐Aldrich, St. Louis, MO, USA). Bone marrow (BM) mononuclear cells from AML patients were isolated by Ficoll density gradient centrifugation (GE Healthcare, Uppsala, Sweden) and cultured in StemSpan SFEM (Stemcell Technologies, Vancouver, Canada) supplemented with human recombinant interleukin‐3 (IL‐3, PeproTech, Rocky Hill, NJ, USA), stem cell factor (SCF, PeproTech), and interleukin‐6 (IL‐6, PeproTech) at final concentrations of 10 ng/mL. All the patients gave informed consent. Normal human CD34^+^ HSPCs were isolated from umbilical cord blood (UCB) and enriched by an immunomagnetic positive selection kit (Stemcell Technologies). Bort (MCE, Princeton, NJ, USA), Palbociclib (MCE), and Bay 11‐7082 (MCE) were dissolved in dimethyl sulfoxide (DMSO) and kept at −20°C until used. All procedures in our studies involving human participants were following the Ethics Committee of the First Affiliated Hospital of Wenzhou Medical University and the Declaration of Helsinki. The clinical characteristics of AML patients are summarized in Table S1.

### 2.2 Quantitative real‐time PCR (qRT‐PCR)

Total RNA was extracted using TRIzol (Invitrogen, Carlsbad, CA, USA) according to the manufacturer's instruction. After extraction, absorbance at 260/280 nm was measured to assess RNA concentration and quality (DS‐11 spectrophotometer, DeNovix, Wilmington, DE, USA). We used total RNA as a template to synthesize cDNA for qRT‐PCR using ABI 7500 real‐time PCR system (Applied Biosystems, Carlsbad, CA, USA). Human and murine β‐actin were used as internal controls for human and murine samples, respectively. Relative expression was calculated using the 2^‐ΔΔCT^ method. The primer sequences were indicated in Table S2.

### 2.3 Blood smear and histology

Bone marrow cytospins were stained by Wright‐Giemsa staining using standard protocols.^15^ Paraformaldehyde‐fixed paraffin‐embedded sections of spleen and liver tissues were subjected to HE staining by standard protocols.

### 2.4 Chromatin immunoprecipitation (ChIP) analysis

The binding activity of NF ĸB p65 in the *CDK6* gene promoter was examined by ChIP and qRT‐PCR assay using a ChIP assay kit (17‐295, Millipore, Billerica, MA, USA).^16^ Briefly, nuclear extracts were prepared from bort‐treated and untreated leukaemic cells, which were cross‐linked with 1% formaldehyde for 10 min. Chromatin was sonicated to produce 200‐1000 bp DNA fragments. The sonicated samples were centrifuged at 12 000 × *g* at 4°C to transfer the supernatants to new microcentrifuges. Protein A/G Agarose Beads (#9007, CST) was added to the supernatants to reduce the nonspecific background. Then, protein‐DNA complexes were immunoprecipitated with 5 μg of p65 antibody (#19870, Abcam) and non‐relevant rabbit immunoglobulin G (#171870, Abcam) at 4 ℃ overnight with constant rotation. The DNA‐protein cross‐link was reversed by heating at 65°C for 4 hours, and then DNA was purified. Standard PCR reactions were performed with two pairs of different primers (Table S2). For the input control, 1% of the sonicated pre‐clear DNA was saved and purified with the precipitated immune complex at the same time. The fold enrichment was calculated as a percentage relative to the input DNA using the 2^‐ΔΔCt^ method.

### 2.5 Construction of plasmids

The whole coding sequence (CDS) of human *CDK6* (NM_001145306) and murine *Cdk6* (NM_009873) were amplified and inserted in the lentiviral vector pLVX‐puro. Five NF ĸB binding sites (5 × GGGGACTTTCCACT) were directly synthesized and constructed in pProUTR‐Reporter plasmid (HarO Biotech, Shanghai, China) carrying Firefly luciferase (Luc) and Renilla luciferase (RLuc). pCMV‐NF ĸB p65 (NM_02975) was purchased from Sino biological company (HG12054, Beijing, China). The primers for cloning were indicated in Table S2.

### 2.6 Other procedures

Cell proliferation by CCK8, Apoptosis, Cytoplasmic and nuclear extraction, Western blotting, Luciferase activity detection, Colony formation assay, Limiting dilution assays, Flow cytometry analysis, lentivirus production and cell transduction, Primary AML blasts‐xenografted NOD/SCID‐IL2Rγ (NSG) mouse model, MF9‐induced murine leukaemia model, RNA sequencing analysis please see Appendix S1.

### 2.7 Statistical analysis

Unless otherwise specified, results are depicted as the mean ± SD. Statistical analyses were performed using Student's *t*‐test. The *P* values were two‐tailed, and a value *P* < .05 was considered statistically significant. Overall survival (OS) probabilities were estimated by the Kaplan‐Meier method, and differences in survival distributions were compared using the log‐rank test. OS was defined from the date of engraftment to death. All statistical analyses were performed using SPSS 22.0 (SPSS Inc, Chicago, IL, USA).

## 3 RESULTS

### 3.1 Anti‐leukemogenesis ability of bort in MLL‐rearranged leukaemia blasts

**FIGURE 1:**
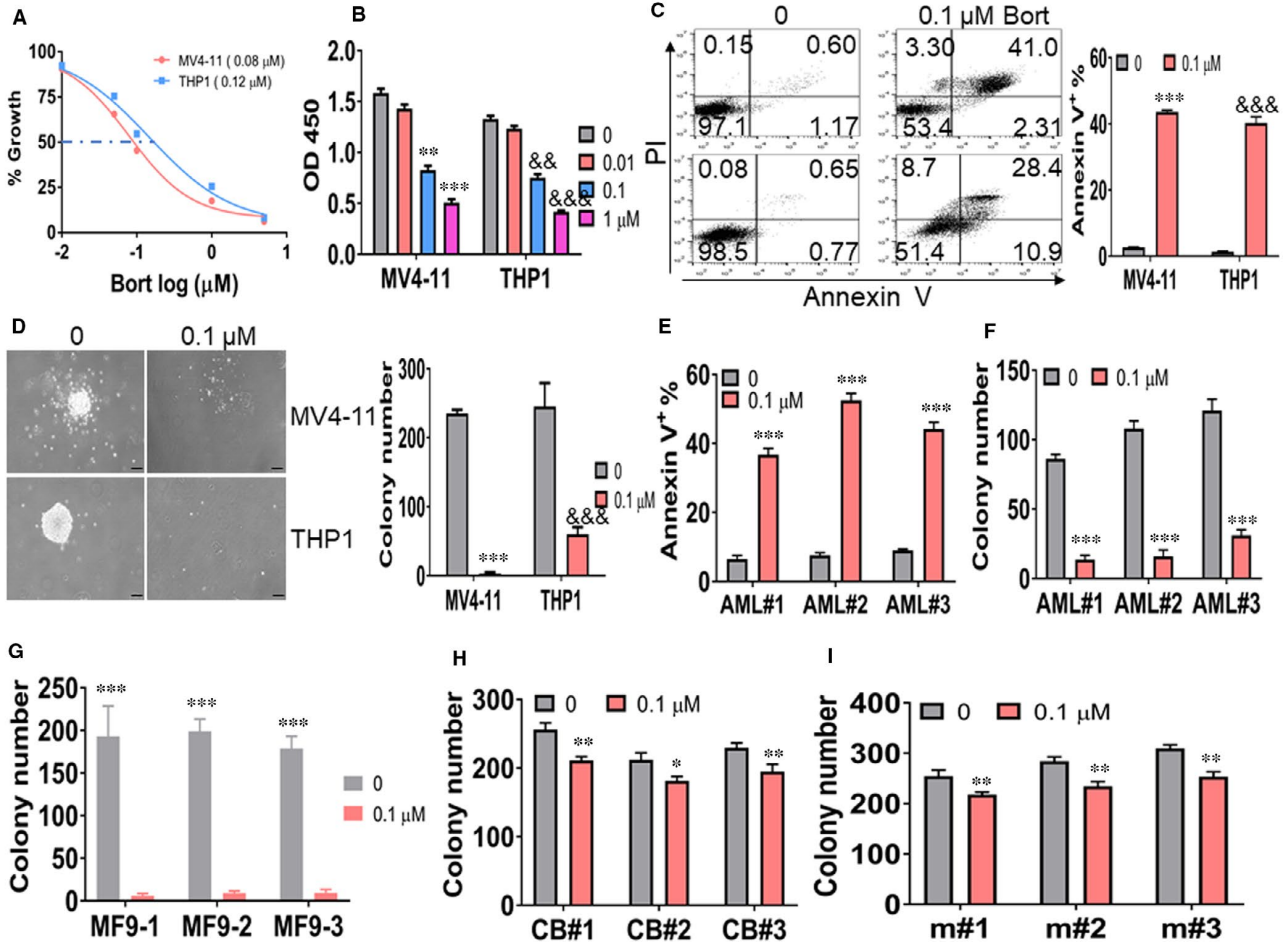
Anti‐leukemogenesis by bortezomib (bort) in leukaemic cells. A, MV4‐11 and THP1 cells were treated with different concentrations of bort for 24 h. Cell growth was assessed by CCK‐8 assay. A 50% inhibitory concentration (IC50) of bort was calculated. B, MV4‐11 and THP1 cells were incubated with indicated concentrations of bort for 24 h. CCK‐8 assay was performed to assess cell proliferation. C, Apoptosis was measured by Annexin V/PI staining in MV4‐11 and THP1 cells, treated with 0.1 µmol/L bort for 24 h. Shown are the representative plots (left) and statistical analysis of Annexin V^+^ cells (right). ^***^ and ^&&&^ *P *< .001 vs untreated cells. (D) MV4‐11 and THP1 cells (2 × 10^3^) were incubated with or without bort (0.1 µmol/L) and were plated on methylcellulose medium. Colony formation was counted after ten days. Shown are representative pictures of colonies (left) and statistical analysis of colony number (right). The bar represents 10 µm, and these images were amplified 100 folds. ^***^ and ^&&&^ *P *< .001 vs untreated cells. (E) Apoptosis was measured in three primary blasts from AML patients with positive MLL‐AF9, which were treated with or without 0.1 µmol/L bort for 24 h. ^***^ *P *< .001 vs untreated cells. (F) CD34^+^ cells (4 × 10^3^) were isolated from BM of three AML patients in Figure 1D, followed by treatment with or without bort (0.1 µmol/L) and plating on methylcellulose medium. Colony formation was counted after ten days. ^***^ *P *< .001 vs untreated cells. G, BM GFP^+^ cells were isolated from MLL‐AF9 (MF9)‐induced leukaemic mice and were treated with or without bort (0.1 µmol/L). GFP^+^ cells (2 × 10^3^) were plated on methylcellulose medium, and colony formation was counted after ten days. ^***^ *P *< .001 vs untreated cells. H, Human CD34^+^ cells (4 × 10^3^) were isolated from three cord blood, followed by treatment with or without bort (0.1 µmol/L) and plating on methylcellulose medium. Colony formation was counted after ten days. (I) Murine c‐Kit^+^ cells were isolated from BM of three normal C57/B6 mice. c‐Kit^+^ cells (4 × 10^3^) were plated on methylcellulose medium treated with or without bort (0.1 µmol/L), and colony formation was counted after ten days

To explore the possible anti‐leukemogenesis ability by bort, we measured the proliferation, apoptosis, and colony formation assay in bort‐treated MV4‐11 and THP1 cells, which carry MF4 and MF9, respectively.^17^, ^18^ We first measured the 50% inhibiting concentration (IC50) of bort and found that IC50 values are 0.08 μmol/L in MV4‐11 and 0.12 μmol/L in THP1 cells at 24 hours (Figure 1A). Bort inhibited the proliferation in a concentration‐dependent manner in MV4‐11 and THP1 cells 24 hours after incubation (Figure 1B). Meanwhile, bort (0.1 μmol/L) substantially induced apoptosis (Figure 1C). To explore if bort inhibits the activity of leukaemic progenitor cells, we evaluated colony formation in leukaemic cells. Above 80% of colony formation was reduced by bort treatment in MV4‐11 and THP1 cells (Figure 1D). To further investigate the anti‐leukemogenesis in leukaemic blasts from AML patients, three primary AML blasts with MF9 rearrangement were treated with bort to assess the apoptosis and colony number. Bort substantially induced apoptosis in primary blasts (Figure 1E) and resulted in a 70%‐80% reduction of colony formation (Figure 1F) in all three primary blasts. We then isolated BM GFP^+^ cells from MF9‐transformed leukaemic mice for counting colony formation. As expected, bort almost wholly eradicated colony formation in GFP^+^ cells from three leukaemic mice (Figure 1G). Finally, we assessed the possible inhibitory effects of bort on normal human and murine HSPC. Three independent CD34^+^ HSPCs from CB and three independent c‐Kit^+^ progenitor cells from BM of normal C57BL/6J mice were treated with or without bort. By contrast, bort slightly reduced colony formation in normal human (Figure 1H) and murine progenitor cells (Figure 1I). These results demonstrate that bort effectively eradicates leukaemic cells but has little effect on normal progenitor cells.

### 3.2 Anti‐self‐renewal ability of LSC by bort in MF9‐transformed leukaemic mice

**FIGURE 2:**
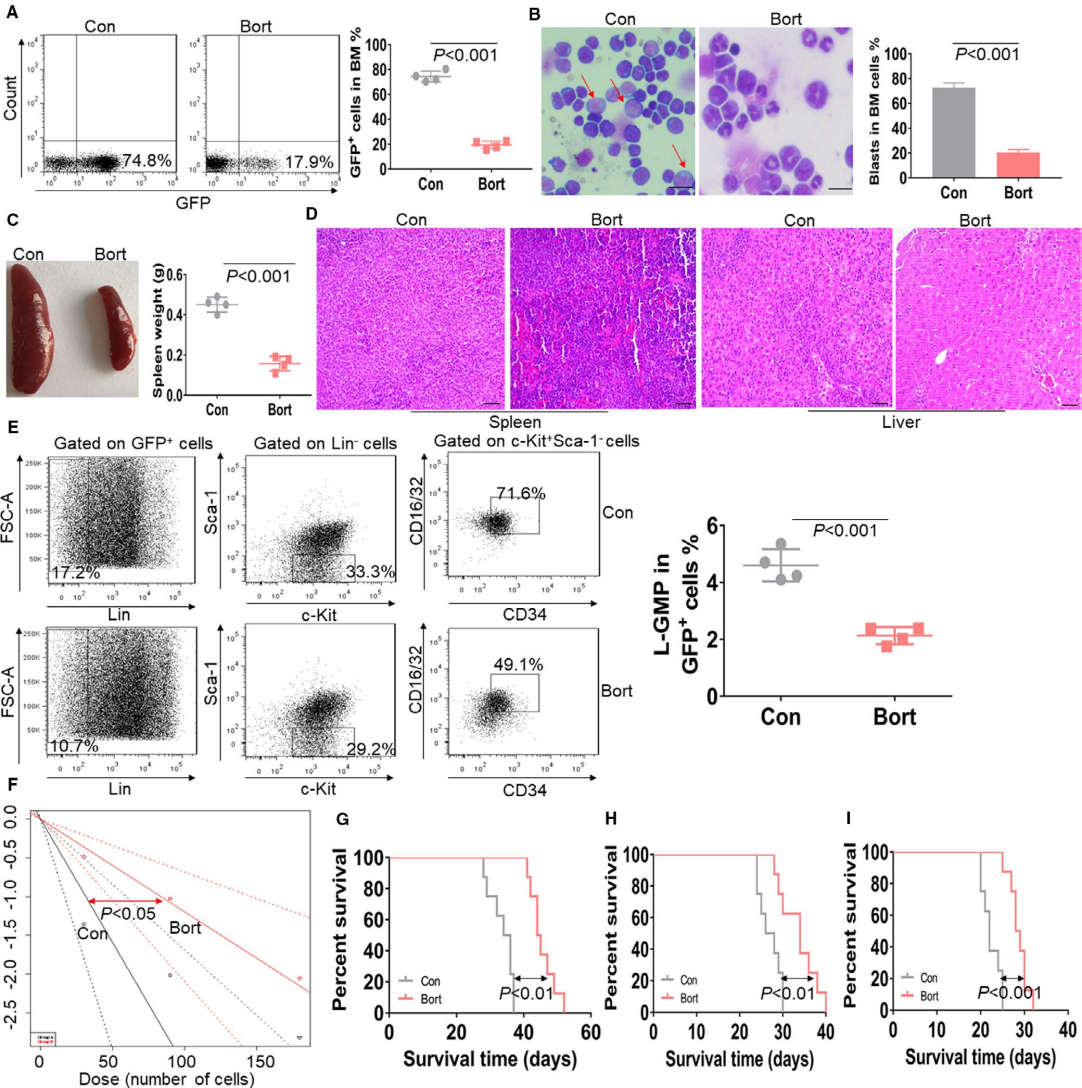
Anti‐self‐renewal and leukemogenesis abilities by bort in MLL‐AF9‐transformed mice model. A, GFP^+^ cells were measured in BM mononuclear cells isolated from bort‐treated (n = 4) or not‐treated MLL‐AF9‐transformed mice (n = 4), when the control mice developed full‐blown leukaemia. Shown are the representative plots (left) and statistical analysis of GFP^+^ cells (right). B, A representative image of BM smear by Wright‐Giemsa stain in MLL‐AF9‐transformed mice treated with or without bort (left) and statistical analysis of average leukaemia blasts (right). The bar represents 10 µm, and these images were amplified 200 folds. ** *P* <.01 vs vehicle mice. C, A representative image of the spleen (left) and statistical analysis of spleen weight (right) in the control mice (n = 4) and bort‐treated mice (n = 4). D, The representative images of spleen and liver tissues from the control mice and bort‐treated mice. The bar represents 10 µm. E, The frequencies of L‐GMP cells were measured in the control mice (n = 4) and bort‐treated mice (n = 4). Shown are the representative plots (left) and statistical analysis of L‐GMP cells (right). (F) Limiting dilution assay of BM GFP^+^ cells from control (n = 8) and bort‐treated mice (n = 8). The frequency of L‐GMP cells and P‐value were calculated by L‐calc software. G–I, Overall survival was analysed in the primary BMT (G, n = 8), second BMT (H, n = 8), and tertiary BMT (I, n = 8) of MLL‐AF9‐induced leukaemic mice treated with bort or not

MF9‐transformed murine modelwas then performed to evaluate the anti‐leukemogenesis by bort *in vivo*.^3^ As shown in Figure S1 A, BM GFP^+^ cells were isolated from MF9‐transduced leukaemic mice, followed by transplantation into recipient mice treated with or without bort. The percentage of GFP^+^ cells, which represent leukaemic cells, was first measured in peripheral blood (PB) at 20 days after transplantation. The percentage of GFP^+^ cells was about 5‐fold lower in the bort‐treated mice compared with the control mice (Figure S1 B). GFP^+^ cells in BM were further measured when the untreated mice developed full‐brown leukaemia. As shown in Figure 2A and Figure S1 C, GFP^+^ cells were about 4‐fold lower in the bort‐treated mice than the control mice. Giemsa‐Wright staining indicated that leukaemic blast in BM was reduced in the bort‐treated mice compared with the control mice (Figure 2B). Furthermore, we evaluated the anti‐leukemogenesis by bort in the spleen and liver. Bort treatment led to above 2‐fold lower in the weight of spleen (Figure 2C) and reduced the infiltration of leukaemic cells in the spleen and liver by the histological H&E staining (Figure 2D). Also, bort treatment substantially reduced the GFP^+^ cells in the spleen (Figure S1 C).

To further assess the anti‐self‐renewal ability of LSC by bort, the frequency of L‐GMP (Lin^‐^c‐Kit^+^Sca‐1^‐^CD34^+^/CD16/32^+^) as LSC was measured.^19^ The frequency of L‐GMP in bort‐treated mice was 2‐fold lower than that in control mice (Figure 2E). More importantly, limiting dilution analysis indicated that bort treatment caused a 70% decrease of functional LSC in leukaemic mice than control mice (1 in 83 vs. 1 in 31, Figure 2F and Table S3). Finally, we performed serial BMT assays to explore the anti‐long‐term self‐renewal of LSC by bort. The survival time is significantly longer in primary bort‐treated mice than control mice (Figure 2G). In the secondary BMT assay, BM GFP^+^ cells from bort‐treated and untreated mice were transplanted to recipient mice. The survival time was markedly extended in bort‐treated mice than untreated mice (Figure 2H). Then, we used secondary leukaemic BM blasts as donor cells to performed tertiary mouse BMT. The survival time in bort‐treated mice was significantly prolonged than that in untreated mice (Figure 2I).

### 3.3 Bort presents anti‐leukemogenesis activity in AML blast‐transplanted NSG mice model

**FIGURE 3:**
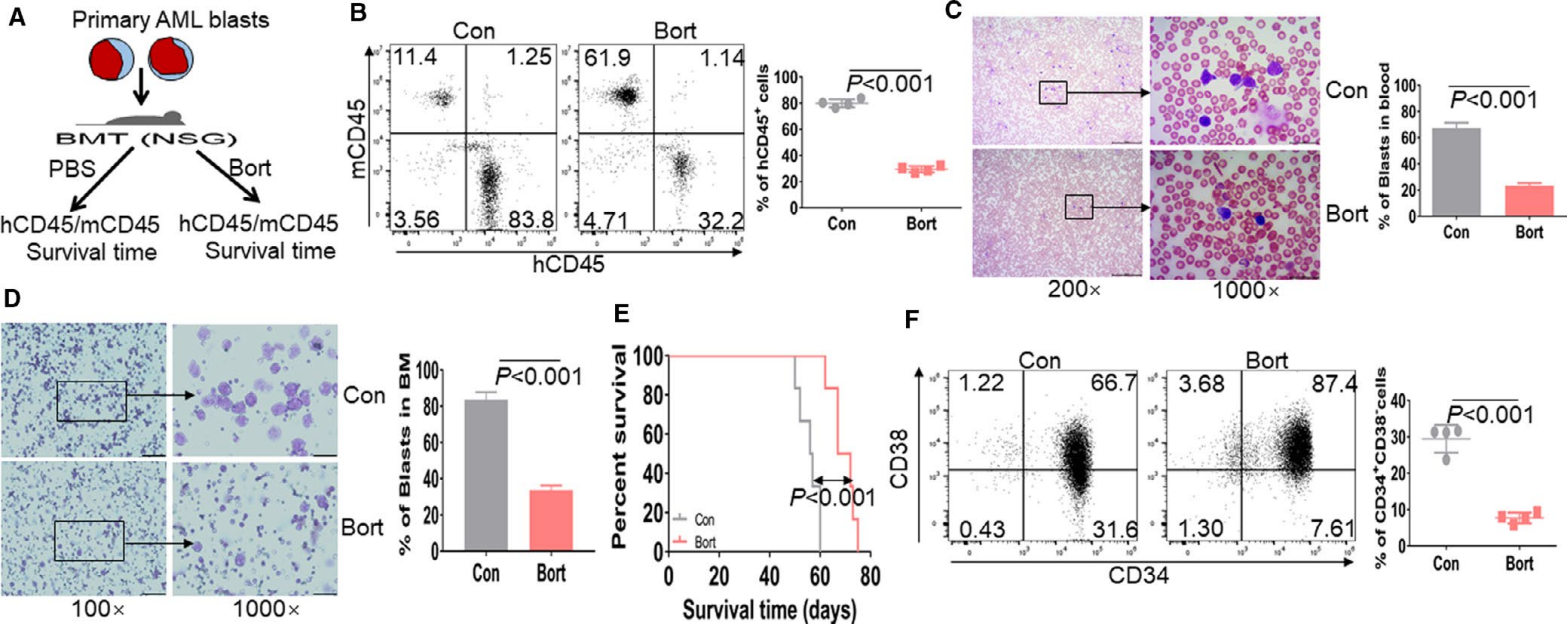
Anti‐leukemogenesis by bort in primary AML blasts‐xenografted mice. A, A schematic outline of the in vivo experiment using AML blasts‐xenografted NSG mice treated with bort or not. B, Human CD45 cells (hCD45) and murine CD45 (mCD45) were measured in peripheral blood from AML blasts‐transplanted NSG mice treated with (n = 4) or without bort (n = 4). Shown are the representative plots (Left) and statistical analysis of hCD45^+^ cells/(hCD45^+^+mCD45^+^) (Right). C and D, Leukaemic blasts were evaluated by Wright‐Giemsa stain in peripheral blood (C) and BM (D) when the control mice became moribund. The bar represents 10 µm. E, Overall survival for THP1‐xenografted NSG mice treated with (n = 6) or without bort (n = 6). F, CD34^+^ CD38^‐^ cells gated on hCD45 were measured in peripheral blood from AML blasts‐transplanted NSG mice treated with (n = 4) or without bort (n = 4). Shown are the representative plots (Left) and statistical analysis of CD34^+^ CD38^‐^ cells (Right)

To further investigate the anti‐leukemogenesis in human AML, we transplanted primary AML blasts bearing MF9 in NSG mice (Figure 3A). The percentage of human CD45 (hCD45)/murine CD45 (mCD45) plus hCD45 representing the chimerism was measured in peripheral blood from AML blasts‐xenografted NSG mice treated with bort or not. The percentage of hCD45 was decreased by more than 2.6‐fold in blood from the bort‐treated mice than the control mice (Figure 3B). Furthermore, bort substantially reduced the progression of leukaemia blasts in blood (Figure 3C) and BM (Figure 3D) by Wright‐Giemsa staining and significantly extended the survival time (Figure 3E; *P* < .01). Human LSC is well accepted to be CD34^+^CD38^‐^ cell population, which can reconstitute human AML in immunodeficient mice.^20^ Therefore, we measured the percentage of CD34^+^CD38^‐^ cell population gated by hCD45 and found that CD34^+^CD38^‐^ cell population was about 3.0‐fold lower in the bort‐treated mice than the control mice (Figure 3F).

### 3.4 CDK6 is the potential target of bort

**FIGURE 4:**
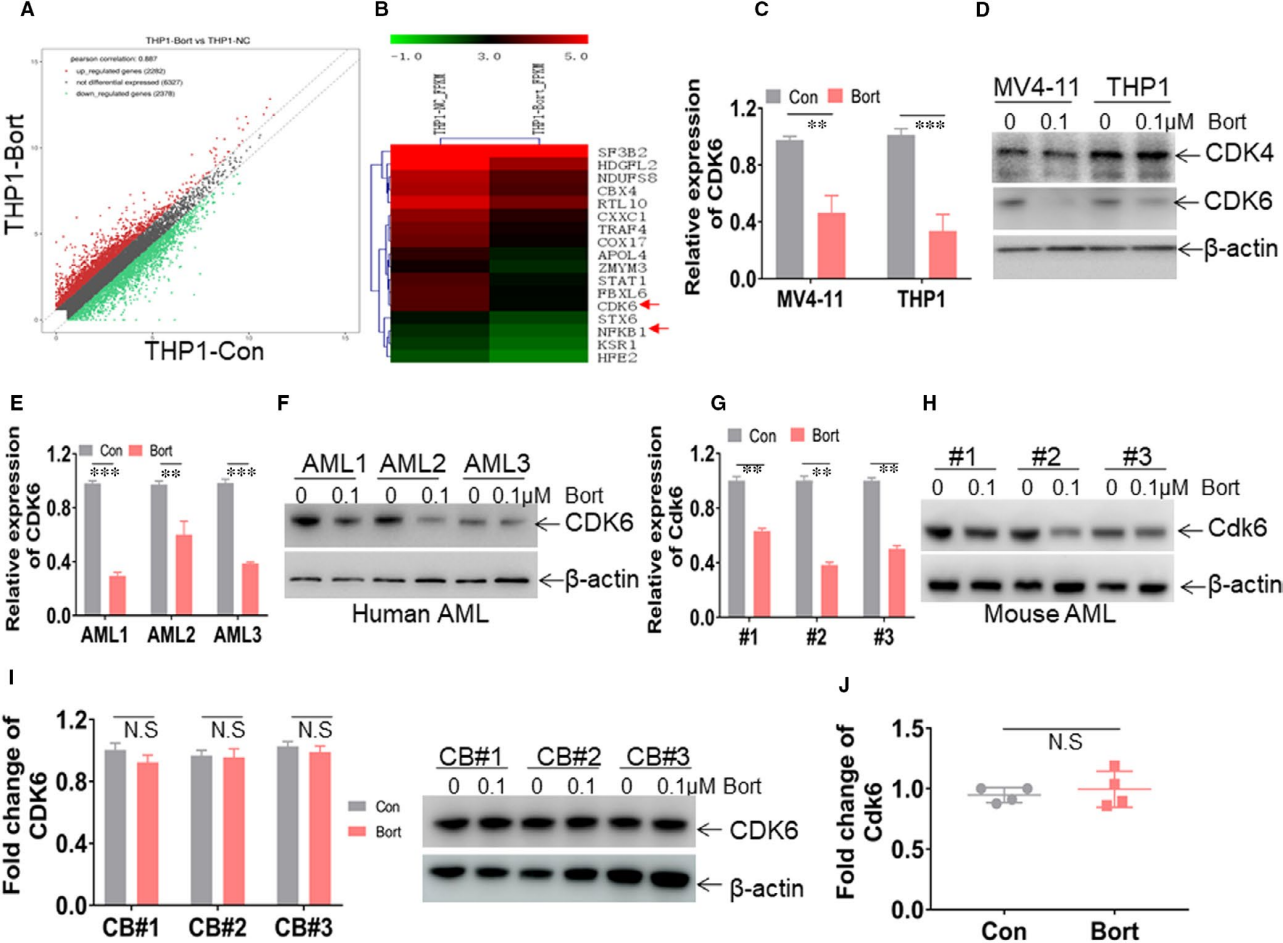
CDK6 is a target by bort. A, RNA sequencing from THP1 cells treated with or without bort was performed for selecting potential target genes by bort. Scatter plots were indicated for the up‐regulated genes (red plots) and down‐regulated genes (green plots) above 2‐fold or below 2‐fold by bort. B, Heatmap representation of down‐regulated genes by bort. Shown is CDK6, which is negatively regulated by bort. C and D, The transcript (C) and protein expressions (D) of CDK6 were measured in MV4‐11 and THP1 cells treated with bort (0.1 µmol/L) or not for 24 h. ^**^ *P *< .01 and ^***^ *P *< .001 vs untreated cells. (E and F) The transcript (E) and protein expressions (F) of CDK6 were assessed in BM blasts from the same three AML patients in Figure 1D. ^**^ *P *< .01 and^***^ *P*<0.001 vs untreated cells. (G and H) BM GFP^+^ cells were isolated from three MLL‐AF9‐transformed leukaemia mice treated with or without bort for the evaluation of transcript (G) and protein expressions (H) of Cdk6. ^**^ *P *< .01 vs untreated cells. (I) CDK6 transcripts and protein expressions were measured in human CD34^+^ cells from cord blood (CB), treated with or without bort (0.1 µmol/L) for 24 h. (J) Normal C57/B6 mice were intraperitoneally injected with 100 μL PBS as the control group (n = 4) and with bort as the experimental group (n = 4). After treatment for four weeks, *Cdk6* transcripts were measured in BM c‐Kit^+^ cells

We next performed RNA sequencing to explore the potential targets of bort. Bort‐treated and ‐untreated THP1 cells were performed to compare the differential expression of genes. We identified more than 2000 genes, which were differentially expressed after bort treatment (more and less than 2.0‐fold; Figure 4A). Among these differential expressions of genes, *CDK6* was finally selected for further study (Figure 4B) because CDK6, a vital modifier of the cell cycle, is indispensable for the initiation and maintenance of MF9‐rearranged leukaemia. Consistent with results from RNA sequencing, bort treatment resulted in a 60% decrease of *CDK6* in THP1 and MV4‐11 cells (Figure 4C). Besides, bort treatment decreased the protein levels of CDK6, but not its functional homolog CDK4 in THP1 and MV4‐11 cells (Figure 4D). Furthermore, three AML blasts (Table S1) with MF9 rearrangement were treated with bort or not in vitro. The transcript and protein expressions of CDK6 were substantially decreased in all three bort‐treated AML blasts compared with untreated blasts (Figure 4E,F). Also, bort decreased Cdk6 expressions in three independent leukaemic cells from MF9‐transformed leukaemic mice (Figure 4G,H). As CDK6 is required for the proliferation of normal HSPC, we then determined whether bort affects the expression of *Cdk6* in normal human and murine HSPCs. Bort treatment did not affect the transcript and protein expressions of CDK6 in human HSPCs in vitro (Figure 4I). Also, the transcripts of *Cdk6* were assessed in BM c‐Kit^+^ cells from bort‐treated and untreated C57BL/6J mice. Bort treatment did not affect the transcript of *Cdk6* in murine HSPCs in vivo (Figure 4J).

### 3.5 Bort reduces the level of CDK6 by inhibiting NF ĸB p65 recruitment to *CDK6* promoter

**FIGURE 5:**
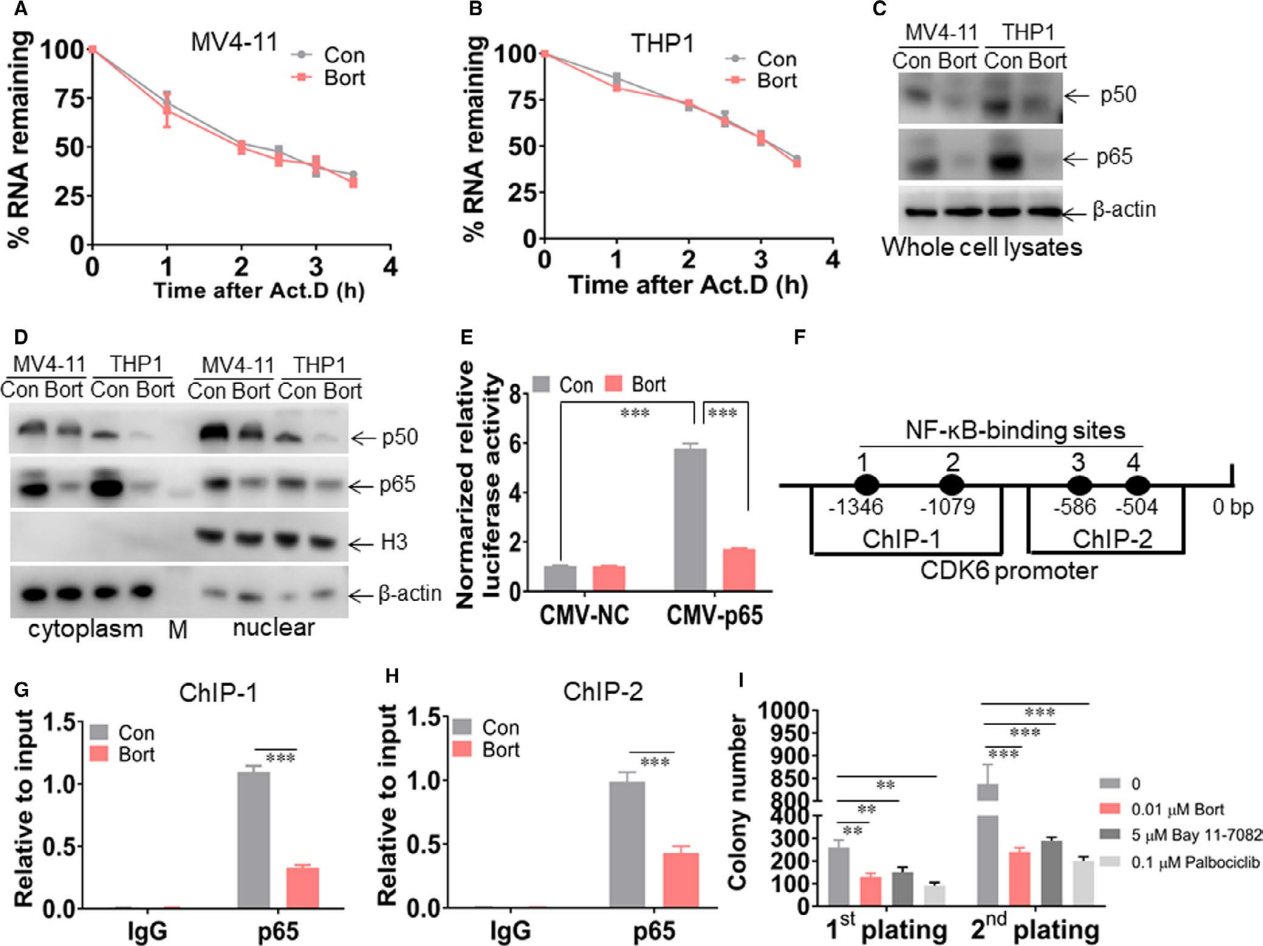
Bort inhibits NF‐ĸB p65 recruitment to *CDK6* promoter. A and B, MV4‐11 and THP1 cells were treated with or without bort at 24 h, followed by incubation of actinomycin D (2 µg/mL) for the indicated times. Cellular mRNA was extracted, and qRT‐PCR was performed to assess the half‐lives of *CDK6* mRNA. C, Western blot for p65 and p50 in the whole‐cell lysates from MV4‐11 and THP1 cells, which were incubated with or without 0.1 µmol/L bort for 24 h. D, Western blot for p65 and p50 in the cytoplasm and nucleus from MV4‐11 and THP1 cells, which were incubated with or without 0.1 µmol/L bort for 24 h. E, 293T cells were transduced with pCMV‐p65 (0.5 µg) or pCMV‐NC (0.5 µg), together with pProUTR‐Reporter plasmid carrying five NF ĸB binding motifs. After transfection for 24 h, 293T cells were treated with or without bort for 24 h. Both firefly and renilla luciferase activities were measured in these cells. Histograms illustrate firefly luciferase activities normalized to renilla luciferase activities. Normalized luciferase activity of NC‐transfected cells was arbitrarily set to 1.0. F, A schematic representation of the CDK6 promoter with four potential NF‐ĸB‐binding sites indicated by a dark oval. ChIP‐1 and ChIP‐2 represent the sequence for different primers. G and H, Soluble chromatin from THP1 cells treated with or without bort was immunoprecipitated with an anti‐p65 antibody. Immunoprecipitated DNA was analysed by qPCR. ChIP‐1 and ChIP‐2 represent two different primers to amplify immunoprecipitated DNA. (I) THP1 cells (2 × 10^3^) incubated with bort (0.01 μmol/L), CDK6 inhibitor Palbociclib (0.1 μmol/L), and NF‐ĸB inhibitor Bay 11‐7082 (5.0 μmol/L) were seeded in methylcellulose medium. Colony formation was counted after ten days. After first plating, untreated and treated THP1 cells (1 × 10^3^) were seeded in methylcellulose medium for second assay. ^**^ *P *< .01 and ^***^ *P *< .001 vs untreated cells

To explore the underlying mechanism by which bort regulates *CDK6* expression, we first measured the half‐life of *CDK6* mRNA to exclude whether bort shortens the half‐life of *CDK6* mRNA, resulting in the down‐regulation of CDK6 in leukaemic cells. Bort‐treated and untreated cells were incubated with actinomycin D (Act. D) for different times. As indicated in Figure 5A,B, bort did not modulate the half‐life of *CDK6* mRNA in MV4‐11 and THP1 cells. Therefore, we hypothesized that bort reduces the expression of *CDK6* mRNA by inhibiting the synthesis of *CDK6* mRNA.

Previous research indicated that transcription factors, such as *NF‐ĸB*, positively regulate the expression of *CDK6* by direct binding to its promoter.^21^ Bort has been reported to suppress NF‐ĸB‐dependent transcription in cutaneous T‐cell lymphoma.^22^ Besides, bort prevented the translocation of NF‐ĸB into the nucleus, leading to the down‐regulation of p‐glycoprotein in leukaemic cells.^23^ Therefore, we hypothesized that bort reduces the level of CDK6 by preventing NF‐ĸB recruitment to *CDK6* promoter. Two major subunits in the NF‐ĸB family (p65 and p50) were measured in the cytoplasm and nucleus. As expected, bort attenuated the levels of p65 and p50 in whole‐cell lysates (Figure 5C), as well as the cytoplasm and nucleus (Figure 5D).

To further determine whether bort attenuates the binding ability of NF‐ĸB p65 on decameric DNA motifs, we assessed luciferase activities in 293T cells. These cells were transduced with pProUTR‐Reporter plasmid carrying five NF‐ĸB binding sites, together with pCMV‐p65 or pCMV‐NC. After transfection for 24 hours, 293T cells were treated with or without bort. As indicated in Figure 5E, transient overexpression of pCMV‐p65 substantially increased the luciferase activity in comparison to pCMV‐NC. While bort treatment decreased NF‐ĸB p65‐induced luciferase activity by about 3‐fold. Then, ChIP analysis for p65 was used to assess whether bort attenuates NF‐ĸB p65 recruitment to *CDK6* gene promoter. Using the PROMO site (http://alggen.lsi.upc.es/cgi‐bin/promo_v3/promo),^24^ we found four potential NF‐ĸB p65 binding motifs (Figure 5F). Two independent ChIP primers were designed to amplify the p65‐binding motifs. As indicated in Figure 5G,H, relative enrichment in the bort‐treated cells was 2‐fold lower than that in the control cells amplified by primer one and primer two, respectively.

Finally, replating assays were performed to investigate the potential anti‐self‐renewal ability of bort, NF‐ĸB inhibitor Bay 11‐7082,^25^ and CDK6 inhibitor Palbociclib.^26^ As indicated in Figure 5I, bort, Palbociclib, and Bay 11‐7082 substantially reduced colony formation in first and second replating assays, indicating that NF‐ĸB inhibitor has similar effects as well as bort and bort presents anti‐self‐renewal ability via NF‐ĸB/CDK6 signalling pathway.

### 3.6 Overexpression of CDK6 partially rescues bort‐mediated anti‐leukemogenesis

**FIGURE 6:**
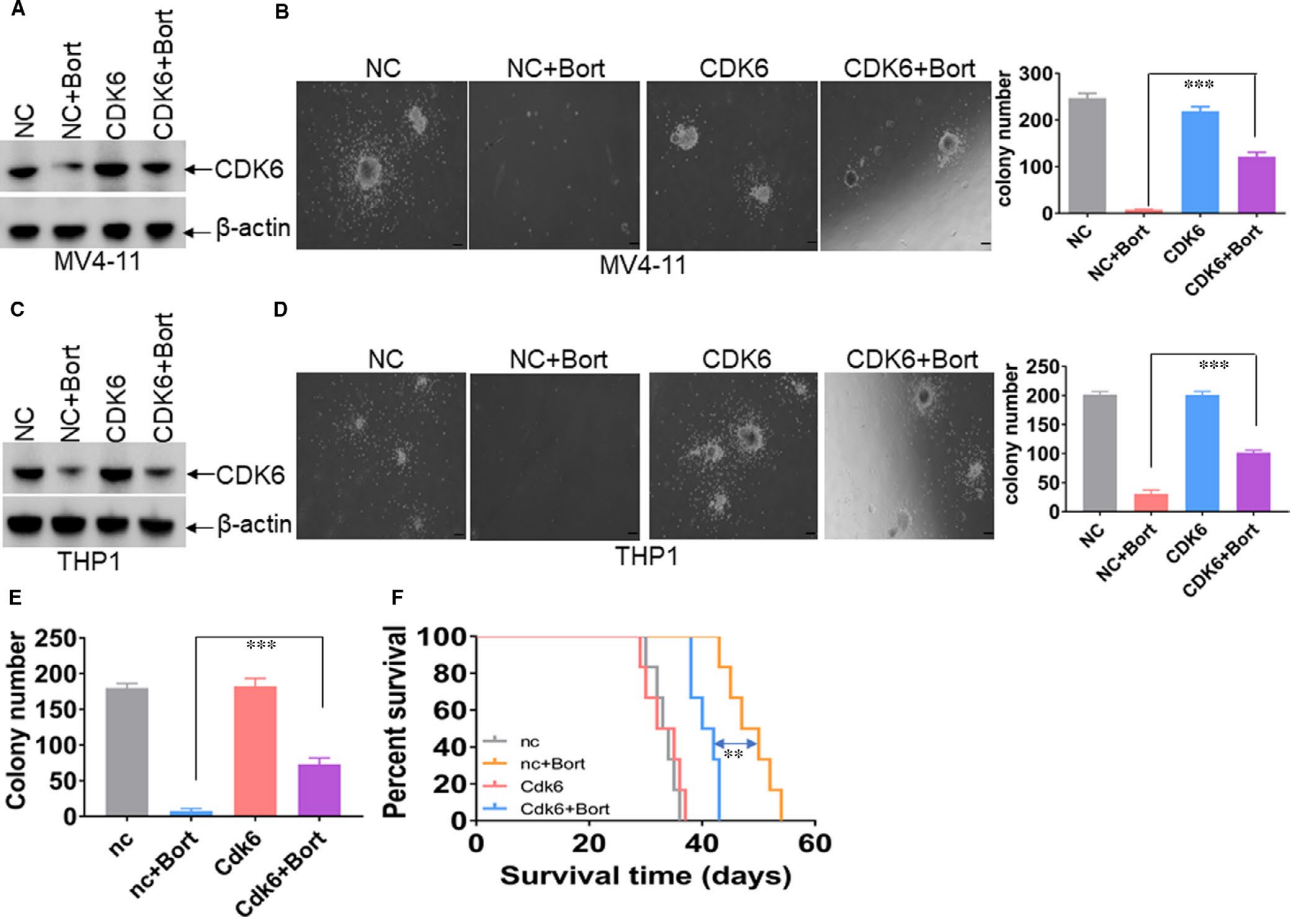
Overexpression of CDK6 partially blocks bort‐induced anti‐leukemogenesis. A and C, Western blot was performed to measure the protein expressions of CDK6 in MV4‐11 and THP1 cells, which were transduced with lentiviral vector overexpressing CDK6 (LVX‐CDK6) or negative control (LVX‐NC), followed by puromycin selection. B and D, Colony formation was counted in CDK6‐or NC‐transduced MV4‐11 (2,000) and THP1 cells (2,000), followed by the treatment of bort (0.1 µmol/L) or not. *** *P* <.001 vs untreated cells. E, BM GFP^+^ cells were isolated from MLL‐AF9‐transformed mice and then were transduced with lentiviral vector overexpressing Cdk6 or NC, followed by puromycin selection. Colony formation was counted in Cdk6‐or nc‐transduced GFP^+^ cells (2,000), which were treated by bort (0.1 µmol/L) or not. *** *P* <.001 vs untreated cells. F, The same amounts of Cdk6‐or nc‐transduced GFP^+^ cells plus a radioprotective dose of whole BM cells were transplanted in lethally irradiated C57BL/6J mice, which were intraperitoneally injected with bort or not (n = 6 for each group). Overall survival time was counted in Cdk6‐or nc‐transduced leukaemic mice treated with or without bort. ** *P* < .01 vs untreated cells

To evaluate whether *CDK6* is an important target of bort, we transduced leukaemic cells with lentiviral vector LVX‐CDK6 overexpressing human CDK6 or empty control vector (NC). CDK6 expression was increased in LVX‐CDK6‐overexpressed leukaemic cells compared with negative control cells (Figure 6A,C). Colony formation was then measured to evaluate the essential role of CDK6 in bort‐induced anti‐leukemogenesis. The decrease of colony formation by bort was partially restored by the overexpression of CDK6 in MV4‐11 and THP1 cells (Figure 6B,D). Furthermore, BM GFP^+^ blasts from MF9‐induced leukaemic mice were transduced with LVX‐Cdk6 or LVX‐nc and treated with bort for colony formation assay. As expected, reduced colony formation by bort was partially blocked by the overexpression of Cdk6 (Figure 6E). Finally, BM GFP^+^ blasts transduced with Cdk6 or nc were transplanted in recipient mice, followed by bort treatment or not. Overexpression of Cdk6 in part prevented the prolonged survival time in MF9‐transformed leukaemic mice treated by bort (Figure 6F).

## 4 DISCUSSION

**FIGURE 7:**
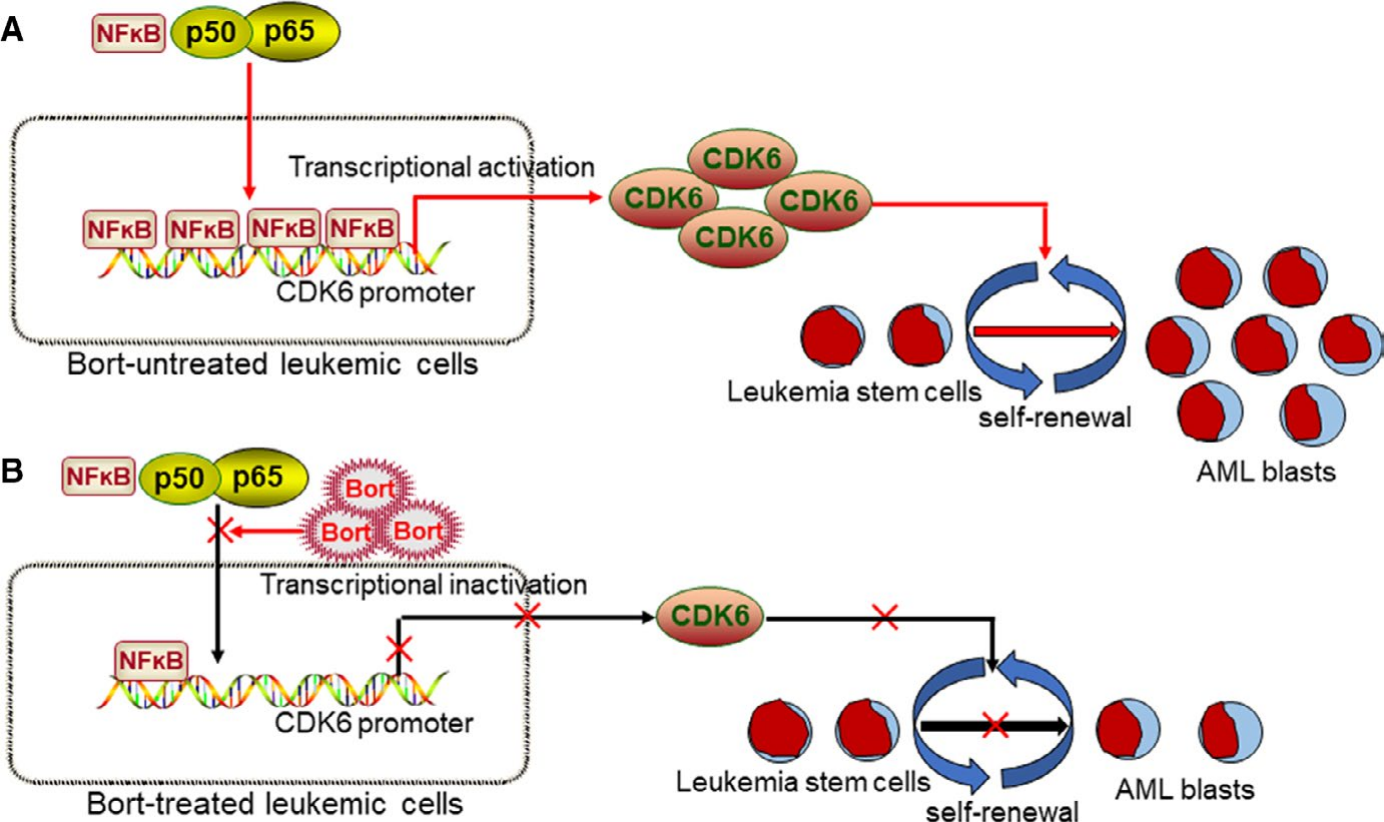
Bort reduces the expression of CDK6 by inhibiting NF ĸB p65 recruitment to CDK6 promoter. A, Without bort, NF ĸB p65 is recruited to CDK6 promoter to activate the expression of CDK6 in leukaemic cells. CDK6 facilitates the proliferation and self‐renewal of LSC. B, Bort treatment inhibits NF ĸB p65 recruitment to CDK6 promoter, resulting in the reduction of CDK6 and finally suppressing the proliferation and self‐renewal of LSC

Bort has been used as a first‐line drug for MM and lymphoma. However, few studies have evaluated the therapeutic efficacy of bort in AML with MLL rearrangements. In our study, we investigated the potential anti‐LSC ability of bort in MLL‐rearranged AML and identified *CDK6* as a target of bort. Without bort, NF ĸB p65 is recruited to *CDK6* promoter to activate the expression of CDK6 in leukaemic cells (Figure 7A). Bort treatment reduced the level of CDK6 by inhibiting NF ĸB p65 recruitment to *CDK6* promoter (Figure 7B). CDK6 is required for the maintenance and development of MLL‐rearranged AML,^14^ and overexpression of CDK6 partially rescued bort‐mediated anti‐leukemogenesis, indicating that CDK6 plays an essential role in the bort‐mediated anti‐leukemogenesis activity. Most importantly, bort possessed a significant selectivity against LSC over normal HSPC, and bort did not affect the expression of CDK6 in normal human and murine HSPC. In conclusion, our results reveal that bort might be a candidate drug for the clinical treatment of AML patients with MLL rearrangements.

Bort presents anti‐cancer activity by reversibly targeting the catalytic 20S core of the proteasome. One of the underlying mechanisms consists of blocking the translocation of NF ĸB to the nucleus and suppressing NF ĸB‐DNA‐binding activity, finally leading to the decreased expression of NF ĸB‐dependent anti‐apoptotic genes.^27^ Our results also indicated that bort attenuated the binding activity of p65 in the *CDK6* gene promoter, finally reducing the expression of CDK6 in leukaemic cells. Although bort is first approved for the treatment of MM and mantle cell lymphoma, several studies have indicated its application in AML. For example, bort displays a high sensitivity in AML patients, whose FAB subtypes are M4 and M5.^28^ This result is consistent with our studies because MF9 mainly occurs in AML patients with M5 subtype.^29^ Besides, bort kills leukaemic blasts independent of the inhibition of ubiquitin‐proteasome signalling. For example, bort induces the cell death of acute promyelocytic leukaemia (APL) by the excessively accumulating *PML‐RARα* fusion gene, followed by the augment of endoplasmic reticulum (ER) stress.^30^ Autophagy induced by bort facilitates the cytotoxic effects in AML cells.^8^, ^31^ In addition, bort induces lysosomal degradation of C‐KIT protein^32^ and induces the degradation of FLT3‐ITD in an autophagy‐dependent manner,^33^ suggesting that bort might has the clinical therapeutic application in C‐KIT‐driven AML and AML patients with positive FLT3‐ITD mutation. Therefore, these results indicate the complicated molecular mechanism by which bort exhibits anti‐leukaemia ability.

As an essential cell cycle regulator, CDK6 and D‐type cyclins facilitate cell proliferation. Recently, studies have indicated that CDK6 is required for the maintenance and development of MLL‐driven AML^14^ and acute lymphoblastic leukaemia (ALL).^17^ Additionally, CDK6 facilitates the development of myeloproliferative neoplasm by increasing cytokine production and activating LSC.^34^ However, these noncanonical functions of CDK6 are not related to the control of cell cycle. Specific inhibition of CDK6 by palbociclib suppresses the self‐renewal ability of leukaemia‐initiating cells in MLL‐driven AML.^14^ Ribociclib, another CDK6 kinase inhibitor used in the clinic, induces the arrest of cell cycle in G1 phase, and enhances glucocorticoid sensitivity in B‐ALL.^35^ In addition, combined inhibition of CDK6 and BCL2 substantially suppressed colony formation and reduced survival of Ph^+^ ALL cells compared with single inhibition of CDK6 or BCL2.^36^ However, considering that CDK6 regulates cell cycle progression, pharmacologic inhibition of CDK6 might cause severe off‐target effects in normal HSPC. Bort only inhibits the expression of CDK6 in leukaemic cells but not in normal HSPCs. Furthermore, bort has little effect on normal HSPC. Therefore, our results suggest that bort‐induced inhibition of CDK6 provides a new therapeutic schedule in leukaemic blasts.

Despite many attempts to understand the molecular mechanism of MLL‐rearranged leukaemia, effective therapy for this type of AML has still lacked. MLL fusion proteins are resistant to ubiquitin‐proteasome‐mediated degradation due to the diminished interactions with E3 ligases Skp2 and Cdc20,^37^ leading to the stabilization and onset for MLL‐rearranged leukaemia. Therefore, the direct degradation of MLL rearrangements through proteasome signalling is difficult in clinical application. Alternative therapy is to target the critical and necessary downstream genes of MLL rearrangements. CDK6 is not only a critical cell cycle regulator but also is a critical downstream gene of MF9. Our results indicated that bort inhibits CDK6 expression via NF ĸB signalling. Therefore, it is interesting to explore whether combined applications of bort, NF kB inhibitors, and CDK6 inhibitors contribute to the clinical intervention of MLL‐rearranged leukaemia.

Although a single‐agent phase I study of bort in children with recurrent/refractory leukaemia demonstrates limited haematologic improvements,^38^, ^39^ combined usage with bort and other compounds indicates a strong anti‐leukaemia activity against AML and provides the potential clinical usage. For example, combination treatment with bort and valproic acid inhibits proliferation and induces apoptosis in AML/MDS.^40^ The addition of bort to standard arsenic trioxide therapy is safe and effective for APL patients at relapse.^41^ Also, the combination therapy of bort, idarubicin, and cytarabine^42^ improves the overall survival in AML in a good safety profile. Therefore, further studies are required to investigate the combined usage with bort and other compounds for AML.

In conclusion, our results indicate that bort presents anti‐leukemogenesis and anti‐self‐renewal activity of LSC in leukaemic cells with MLL rearrangements. Mechanistically, bort reduces the level of CDK6 by inhibiting NF ĸB recruitment to *CDK6* promoter. As the requirement for CDK6 is restricted to the condition of stress, such as oncogenic stress, inhibition of CDK6 might not affect normal haematopoiesis. Also, bort has little side effects against normal HSPC. Therefore, our results first report that CDK6 is a new target for bort through NF ĸB‐dependent manner. Single usage of bort or the combined usage of bort and chemotherapy drugs might provide a new therapeutic strategy for AML patients with MLL rearrangements.

## CONFLICT OF INTEREST

The authors declare that they have no competing interests.

## AUTHOR CONTRIBUTIONS

**Bin Zhou:** Data curation (lead); Methodology (equal). **Yaqian Qin:** Conceptualization (equal); Methodology (equal). **Jingying Zhou:** Conceptualization (equal); Data curation (equal); Methodology (equal). **Jichen Ruan:** Data curation (equal); Formal analysis (equal); Methodology (equal). **Fang Xiong:** Data curation (equal); Methodology (equal). **Jinglai Dong:** Conceptualization (equal); Data curation (equal); Methodology (equal). **Xingzhou Huang:** Methodology (equal). **Zhijie Yu:** Data curation (equal); Methodology (equal). **Shenmeng Gao:** Project administration (lead); Writing‐original draft (lead); Writing‐review & editing (lead).

## Supporting information

Fig S1

Fig S2

Table S1

Table S2

Table S3

Appendix S1

## Data Availability

All data generated or analysed during this study are included in this article.
