# Supplementary figures and images for: Bortezomib suppresses self‐renewal and leukemogenesis of leukemia stem cell by NF‐ĸB‐dependent inhibition of CDK6 in MLL‐rearranged myeloid leukemia
Source: J Cell Mol Med. 2021 Feb 17;25(6):3124–35. doi: 10.1111/jcmm.16377 (PMC7957264; doi:10.1111/jcmm.16377)

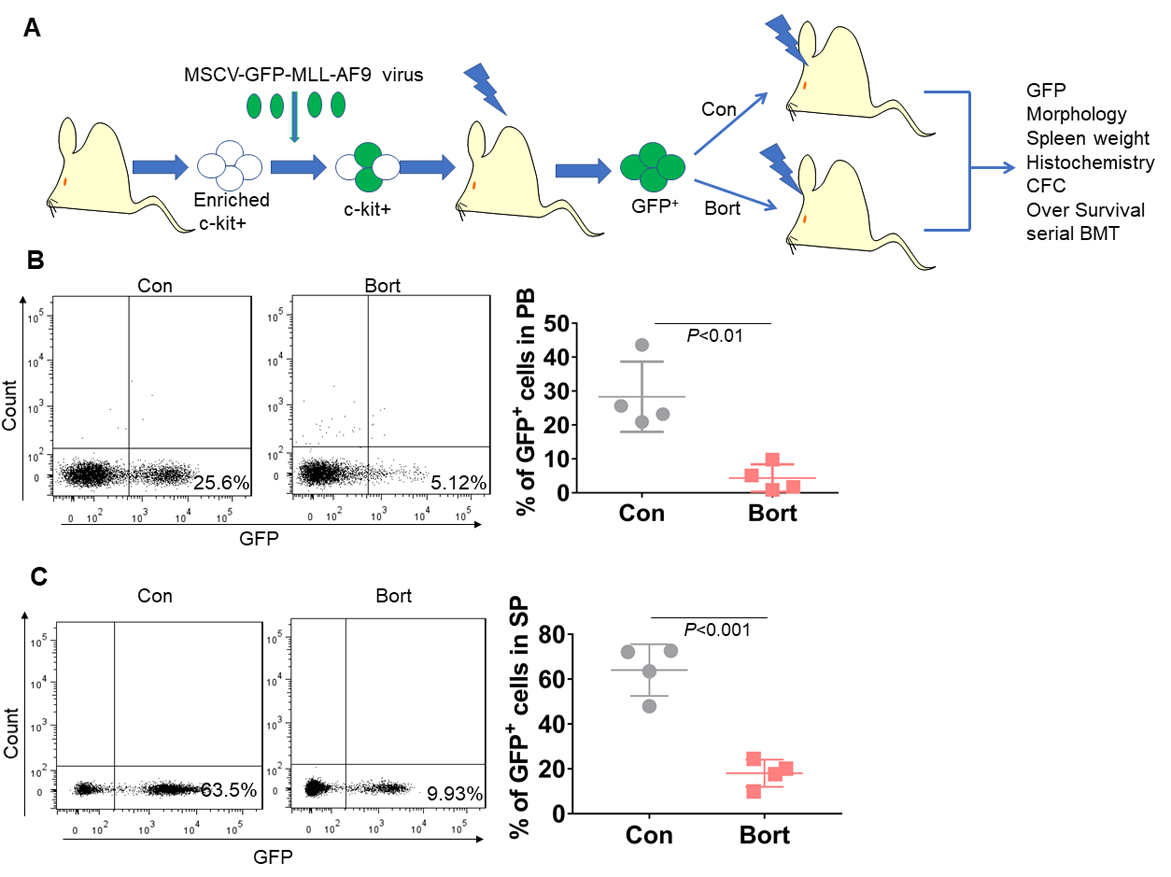

Supplement: Supplementary file 1 — Fig S1 [file JCMM-25-3124-s001.tif]

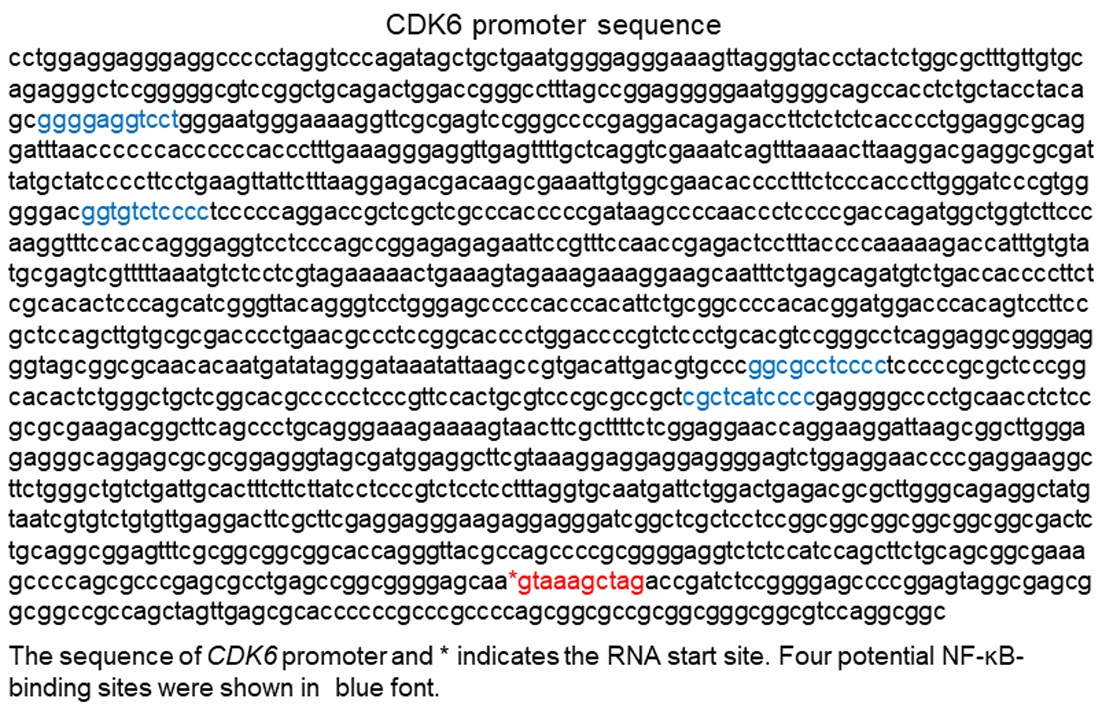

Supplement: Supplementary file 2 — Fig S2 [file JCMM-25-3124-s006.tif]
