# Supplementary material for: Bortezomib suppresses self‐renewal and leukemogenesis of leukemia stem cell by NF‐ĸB‐dependent inhibition of CDK6 in MLL‐rearranged myeloid leukemia
Source: J Cell Mol Med. 2021 Feb 17;25(6):3124–35. doi: 10.1111/jcmm.16377 (PMC7957264; doi:10.1111/jcmm.16377)
Supplement: Supplementary file 3 — Table S1 [file JCMM-25-3124-s002.docx]

**Table S1. Clinical characteristics of AML patients**

| Num | Sex | Age (y) | FAB subtype | Karyotype | Blasts % |
| --- | --- | --- | --- | --- | --- |
| #1 | M | 65 | M5 | 46, XY+ t(9; 11) (p22; q23) | 71 |
| #2 | M | 42 | M5 | 46, XY+ t(9; 11) (p22; q23) | 85 |
| #3 | F | 55 | M5 | 46, XX+ t(9; 11) (p22; q23) | 80 |
