# Supplementary material for: Bortezomib suppresses self‐renewal and leukemogenesis of leukemia stem cell by NF‐ĸB‐dependent inhibition of CDK6 in MLL‐rearranged myeloid leukemia
Source: J Cell Mol Med. 2021 Feb 17;25(6):3124–35. doi: 10.1111/jcmm.16377 (PMC7957264; doi:10.1111/jcmm.16377)
Supplement: Supplementary file 4 — Table S2 [file JCMM-25-3124-s003.docx]

| Genes | Sequences |
| --- | --- |
| CDK6-L | 5′-CCA GAT GGC TCT AAC CTC AGT-3′ |
| CDK6-R | 5′-AAC TTC CAC GAA AAA GAG GCT T -3′ |
| β-actin-L | 5′-TGG CAT CCA CGA AAC TAC CT-3′ |
| β-actin-R | 5′-CGT ACA GGT CTT TGC GGA TG-3′ |
| β-actin-L (mouse) | 5′-GGC TGT ATT CCC CTC CAT CG-3′ |
| β-actin-R (mouse) | 5′-CCA GTT GGT AAC AAT GCC ATG T-3′ |
| Cdk6-L (mouse) | 5′-GGC GTA CCC ACA GAA ACC ATA-3′ |
| Cdk6-R (mouse) | 5′-AGG TAA GGG CCA TCT GAA AAC T-3′ |
| *pLVX-CDK6-L | 5′-CGG AAT TCA TGG AGA AGG ACG GCC TGT G-3′ |
| *pLVX-CDK6-R | 5′-CGG GAT CCT CAG GCT GTA TTC AGC TCC G-3′ |
| *pLVX-Cdk6-L | 5′-CGG AAT TCA TGG AGA AGG ACA GCC TGA-3′ |
| *pLVX-Cdk6-R | 5′-CGG GAT CCT CAG GCT GTG TTC AGC TCC G-3′ |
| ChIP1-CDK6-L | 5′-AGG TCC CAG ATA GCT GCT GA-3′ |
| ChIP1-CDK6-R | 5′-GAA TTC TCT CTC CGG CTG GG-3′ |
| ChIP2-CDK6-L | 5′-GAG CAG ATG TCT GAC CAC CC-3′ |
| ChIP2-CDK6-R | 5′-CCC CTC CTC CTC CTT TAC GA-3′ |

**Table S2: The sequences of primers for qRT-PCR and construction of plasmids**

*Primers for construction of plasmids.
