# Supplementary material for: Bortezomib suppresses self‐renewal and leukemogenesis of leukemia stem cell by NF‐ĸB‐dependent inhibition of CDK6 in MLL‐rearranged myeloid leukemia
Source: J Cell Mol Med. 2021 Feb 17;25(6):3124–35. doi: 10.1111/jcmm.16377 (PMC7957264; doi:10.1111/jcmm.16377)
Supplement: Supplementary file 5 — Table S3 [file JCMM-25-3124-s004.docx]

**Table S3. Limiting dilution assay of MLL-AF9-induced murine leukemia treated with or without bortemozib.**

| Dose | Con (response/total) | Bort (response/total) | *P* value |
| --- | --- | --- | --- |
| 30 | 6/8 | 3/8 |  |
| 90 | 7/8 | 5/8 |  |
| 180 | 8/8 | 7/8 |  |
| LSC frequency | 1 in 31 | 1 in 83 | 0.0187 |

The numbers of response mice mean that the recipient mice develop full-blown leukemia and die within 20 weeks after transplantation.
