# Supplementary material for: Bortezomib suppresses self‐renewal and leukemogenesis of leukemia stem cell by NF‐ĸB‐dependent inhibition of CDK6 in MLL‐rearranged myeloid leukemia
Source: J Cell Mol Med. 2021 Feb 17;25(6):3124–35. doi: 10.1111/jcmm.16377 (PMC7957264; doi:10.1111/jcmm.16377)
Supplement: Supplementary file 6 — Appendix S1 [file JCMM-25-3124-s005.docx]

**Supplementary Material and methods**

**Cell proliferation by CCK8**

Leukemic cells (1×10^5^ cells/ml) were plated in 96-well plates, followed by treatment with or without different concentrations of bort for 24 h. Then, 10 μl of CCK-8 solution (Dojindo, Kumamoto, Japan) was pipetted in 96-well plates and incubated for 4 h. The absorbance was measured at 450 nm using an MRX II microplate reader (Dynex, Chantilly, VA, USA).

**Apoptosis**

Leukemic cells (2×10^5^/ml) treated with or without bort were plated in six-well plates. Apoptosis was measured by annexin V/PI staining (Invitrogen). Briefly, cells were collected and washed by 1×binding buffer. Leukemic cells were then incubated in 100 μl 1×binding buffer with 5.0 μl Annexin V-APC and 5.0 μl PI for 30 min. Cells were washed and resuspended with 500 μl 1×binding buffer and analyzed by flow cytometry (CytoFLEX LX, Beckman-Coulter, Brea, CA, USA) within 30 m after staining.

**Cytoplasmic and nuclear extraction**

Cytoplasmic and nuclear fractions were extracted by using a commercial kit (Thermo Fisher Scientific, Waltham, MA, USA) according to the manufacturer’s instruction. Briefly, leukemic cells were harvested and then washed by cold PBS. After centrifugation, ice-cold CER I was added to the cell pellet. The tube was vortexed vigorously on the highest setting for 15 s to entirely suspend the cell pellet. Then, ice-cold CER II was added to the tube. After centrifugation for 5 m at 16000×g, the supernatant (cytoplasmic extract) was immediately transferred to a clean pre-chilled tube. The insoluble (pellet) fraction containing nuclei was vortexed for 15 s every 10 m, for a total of 40 m. The supernatant (nuclear extract) fraction was transferred to a clean pre-chilled tube, after centrifugation for 5 m at 16000×g. Cytoplasmic and nuclear extracts were stored at -86°C until use.

**Western blotting**

Western blotting analysis was performed according to the standard procedure. Briefly, cell samples were lyzed in cold radio immunoprecipitation assay (Thermo Fisher Scientific) to extract total protein. Protein concentration was measured by a bicinchoninic acid (BCA) protein assay (Thermo Fisher Scientific). The following antibodies were used: CDK6 (1:2000, #124821, Abcam, Cambridge, MA, USA); CDK4 (1:2000, #108357, Abcam); p65 (1:2000, #16502, Abcam); p50 (1:2000, #ab32360, Abcam); β-actin antibody (1:5000, #6276, Abcam) as an internal control. All primary antibodies were diluted in 1×TBST buffer with 2% bovine serum albumin (BSA). All secondary antibod­ies are conjugated with horseradish peroxidase (HRP). Signals were measured by chemiluminescence reagents (Thermo Fisher Scientific).

**Luciferase activity detection**

pProUTR-Reporter plasmid carrying NF ĸB binding sites and pCMV-NF ĸB p65 were transfected in 293T cells, which were treated with bort (0.1 μM) or not for 24 h. Luc and RLuc activities were measured by the Dual-Luciferase Reporter Assay System (Promega) in cell lysates. The value of relative luciferase activity indicates the Luc activity normalized to that of RLuc for each assay.

**Colony formation assay**

BM GFP^+^ cells were isolated from MLL-AF9-induced mouse leukemia. BM c-Kit^+^ cells as normal HSPCs were isolated from 6-week-old wild-type C57BL/6J mice and enriched by immunomagnetic positive selection kit (Stemcell Technologies). Murine leukemic blasts and normal c-Kit^+^ cells were plated into methylcellulose medium (MethoCult™ GF M3434, Stemcell Technologies) treated with or without bort. Human leukemic CD34^+^ cells were isolated from BM of AML patients, and human normal CD34^+^ cells were isolated from umbilical cord blood. These cells were further enriched by immunomagnetic positive selection kit (Stemcell Technologies) and were seeded into methylcellulose medium (MethoCult^TM^ H4434 Classic, Stemcell Technologies) treated with or without bort. Colonies (>40 cells) were counted on ten days after plating according to manufacturer's protocol.

**Limiting dilution assays**

BM GFP^+^ leukemic cells sorted by flow cytometry were isolated from secondary BMT recipients. Three different doses of donor cells were transplanted into lethally irradiated recipients for each group (n=8). The numbers of recipient mice were counted only when they developed full-blown leukemia and died within 20 weeks post-transplantation. We evaluated the frequency of LSC by software extreme limiting dilution assay (ELDA; http://bioinf.wehi.edu.au/software/elda).

**Flow cytometry analysis**

For immunophenotypic analysis of GMP-like leukemic cells (L-GMP), BM cells were washed and stained with biotin-conjugated lineage markers (CD11b, Gr-1, Ter119, CD3, B220, Mouse Hematopoietic Lineage Biotin Panel, BD PharMingen), followed by incubation with Streptavidin-APC-R700, c-Kit-PE, Sca-1-PE-Cy7, CD34-APC, and CD16/CD32-PerCP-Cy5.5 (all from BD PharMingen). The analysis was performed by CytoFLEX LX (Beckman-Coulter, Brea, CA, USA), and cell sorting was performed by FACS Aria II (Becton Dickinson, Mountainview, CA, USA). Data were analyzed by FlowJo software (Becton Dickinson).

**lentivirus production and cell transduction**

For the production of lentivirus, HEK293T cells (4×10^6^) were plated in a 10 cm dish. After 24 h, constructed plasmids and negative control vector (LVX-NC) together with packaging plasmids (MD2G and PSPA2) were co-transfected into 293T cells using Xfect™ Transfection Reagent (Takara Bio, Tokyo, Japan). The virus was collected from the supernatant at 48 and 72 h after transfection and was further filtered by a 0.45 μm polysulfone filter (Millipore). For the production of leukemic cells overexpressing CDK6, leukemic cells (4×10^5^/ml) were suspended in viral supernatant with 8 μg/ml polybrene (Sigma-Aldrich, St. Louis, MO, USA), followed by the centrifugation at 2000×rpm for 2 h. Puromycin (1 μg/ml, MCE, Princeton, NJ, USA) was added into the supernatant to select positive clones for at least one week.

**Primary AML blasts-xenografted NOD/SCID-IL2Rγ (NSG) mouse model**

Busulfan (30 mg/kg; B2635; Sigma) was intraperito­neally given to eight-week-old NSG mice (Shanghai Model Organisms Center, Shanghai, China) one day before xenotransplan­tation. Primary AML blasts (2×10^6^) were intravenously injected into NSG mice, which were divided into two groups (6 mice per group). One group was intraperito­neally injected with 100 μL PBS as the control group, and another group was intraperito­neally injected with bort (1 mg/kg, once for every three days) in 100 μL PBS as the experimental group. Peripheral blood was extracted from control and bort-treated mice to measure hCD45 (hCD45-APC, BD PharMingen)/mCD45 (mCD45-PE, BD PharMingen) as chimerism when control mice developed full-blown leukemia. Human LSC as CD34^+^CD38^-^ (CD34-PE, CD38-BV421, BD PharMingen) was measured by flow cytometry in peripheral blood. Wright-Giemsa stain was performed in peripheral blood to assess the infiltration of AML blasts. All animal procedures and care are performed according to national and international policies and institutional guidelines of the First Affiliated Hospital of Wenzhou Medical University.

**MLL-AF9-induced murine leukemia model**

BM c-Kit^+^ cells were isolated and enriched by immunomagnetic positive selection kit (Stemcell Technologies) from 8-week-old wild-type C57BL/6J mice and cultured in StemSpan SFEM (Stemcell Technologies), which is supplemented with murine SCF (10 ng/ml, PeproTech), thrombopoietin (TPO, 50 ng/ml, PeproTech), and fms related receptor tyrosine kinase 3 ligand (Flt3 ligand, 50 ng/ml, PeproTech). The c-Kit^+^ cells were retrovirally transduced with MSCV-green fluorescent protein (GFP)-IRES-MLL-AF9. The c-Kit^+^ cells plus radioprotective BM cells (2× 10^5^ for every mouse) were intravenously injected into lethally irradiated C57BL/6J mice (Beijing Vital River Laboratory Animal Technology, Beijing, China). BM GFP^+^ cells were sorted from MLL-AF9-induced mice when these mice developed full-blown leukemia. GFP^+^ blasts (1×10^4^) were intravenously injected into lethally irradiated C57BL/6J mice. These mice were randomly divided into two groups: one group was intraperito­neally injected with 100 μL PBS as the control group and another group was intraperitoneally injected with bort (1 mg/kg, once for every three days) as the experimental group. All animal procedures and care are performed according to national and international policies and institutional guidelines of the ethics committee of the First Affiliated Hospital of Wenzhou Medical University.

**RNA sequencing analysis**

Total RNA was extracted from THP1 cells treated with or without bort by using Trizol reagent (Invitrogen, Carlsbad) according to the manufacturer’s protocol. RNA concentration and quality were measured by DS-11 spectrophotometer (DeNovix). KAPA Stranded RNA-Seq Library Preparation Kit (Illumina) was used to construct the cDNA library as the following: poly-A mRNA was isolated from total RNA by using the NEB-Next Oligo d(T) magnetic beads. Then, mRNA was fragmented into small pieces after treatment with the fragmentation buffer. cDNA was synthesized by random primers, DNA polymerase I, and RNase H. Final cDNA library was constructed through suitable fragments by PCR amplification. Sequencing was performed on Illumina HiSeq 4000 sequencing platform after double-stranded cDNA samples were verified with an Agilent 2100 Bioanalyzer (Agilent Technologies). Image analysis, base calling, and error estimation were carried out by Illumina/Solexa Pipeline. The trimmed reads are mapped to the corresponding reference genome by HISAT2 (version 2.0.4), and StringTie (version 1.2.3) was used to reconstruct the transcriptome. All microarray data are available on the Gene Expression Omnibus public database under accession no. GSE157702
